# Rapid Deterioration of a Cervical Tuberculosis Disease in a Young Immunocompetent Patient: A Case Report

**DOI:** 10.7759/cureus.29472

**Published:** 2022-09-22

**Authors:** Mohammed Khashab, Mahmood A Qoqandi, Mohamed Elkhalifa, Alaa T Alsharif, Abdulaziz AlDakhil, Abeer M Alahmadi, Mohammed Alshehri

**Affiliations:** 1 Department of Surgery, Orthopedic Division, King Abdulaziz Medical City/Ministry of National Guard - Health Affairs, Jeddah, SAU; 2 Department of Surgery, Orthopedic Division, King Abdullah International Medical Research Center, Jeddah, SAU; 3 Department of Surgery, Orthopedic Division, Collage of Medicine, King Saud Bin Abdulaziz University for Health Sciences, Jeddah, SAU; 4 Department of Surgery, Orthopedic Division/Orthopaedic Surgery, King Abdulaziz Medical City/Ministry of National Guard - Health Affairs, Jeddah, SAU; 5 College of Medicine, King Saud Bin Abdulaziz University for Health Sciences College of Medicine, Jeddah, SAU; 6 Orthopedics, Prince Sultan Military Medical City, Riyadh, SAU; 7 Public Health, Ministery of Health, Jeddah, SAU

**Keywords:** spine instability, spine surgery, infection, cervical spine, tuberculosis

## Abstract

Any organ system is prone to extrapulmonary tuberculosis (EPTB) development, including the spine. Spinal TB is a rare involvement, although considered one of the most dangerous forms of skeletal TB (STB).

A 31-year-old man, who is a healthcare worker, presented to the outpatient Orthopedic Spine clinic at King Abdulaziz Medical City-Ministry of National Guard Health Affairs (KAMC-MNGHA) Jeddah, Saudi Arabia, with a complaint of axial neck and upper back pain whose condition deteriorated quickly, necessitating urgent admission for surgical treatment in the form of cervical spine decompression and fusion, in addition to the anti-tuberculosis drug (ATD) scheme.

Cervical TB is a rare spinal disease that supposedly has a slow, insidious progression. The main presenting symptoms of which are axial and/or radicular pain, with a possible neurological deficit(s). In this particular case, the rapid progression of the disease necessitated rapid action.

In spite of what is known about spine TB and its slow progression, the case presented here was beyond our expectations. Treatment planning and urgency should not rely on the known natural history of the disease but rather be tailored to each case individually. This delineates the importance of reporting the quick, unexpected deterioration of our patient’s condition.

## Introduction

Tuberculosis (TB) is considered a leading healthcare issue worldwide and is a major cause of mortality from an individual infectious disease agent [1]. It also remains a known public health concern. In Saudi Arabia, the 2019 World Health Organization Report states that the yearly TB incidence is 10 per 100,000 [2]. Mycobacterium tuberculosis is the most common pathogen causing TB [3]. It is an aerobic bacillus that grows slowly over time. The infection’s primary site can be in the lungs, mediastinal lymph nodes, or any visceral structure such as the gastrointestinal tract, mesentery, or genitourinary system. The bacillus tends to multiply every 15 to 20 hours favorably in an aerobic condition while remaining dormant in the process. Spinal infections are always secondary to hematogenous dissemination of the pathogen from a primary site [4,5]. Spine involvement accounts for half of the skeletal TB infections and constitutes 1-2% of TB-infected patients [5-7]. Here, we report a case of cervical tuberculosis with a severe deterioration of symptoms in a very short period of time as well as radiographic findings that raise more questions than answers. The case report was submitted in accordance with SCARE 2020 criteria [8].

## Case presentation

A 31-year-old gentleman, a Saudi Arabian citizen, and a healthcare worker presented to the outpatient Orthopedic Spine clinic at King Abdulaziz Medical City-Ministry of National Guard Health Affairs (KAMC-MNGHA) Jeddah, Saudi Arabia, with a complaint of axial neck and upper back pain with no radiculopathy or weakness on January 4, 2022. The pain started two months prior and was on and off, causing discomfort but no limitation in daily activities. There was no history of preceding trauma. The history of the present illness did not reveal a feature to explain the sudden worsening of the symptoms. The patient was medically and surgically free, not on any medications, and had received no antibiotics whatsoever. There was no recent history of contact with a patient diagnosed with TB, nor was there a history of ingestion of raw milk. There was no history of recent infections, skin lesions, or sexually transmitted diseases, and the patient denied using illicit intravenous (IV) drugs. There were no red flags for neurological deterioration, nor were there any bowel or bladder symptoms. The patient did not develop constitutional symptoms, and his family history was negative for malignancy but was positive for Pott’s Disease in his sister 14 years ago when she was diagnosed and treated. The review of systems was unremarkable. The patient's examination showed mild cervical paraspinal tenderness and a normal neurological examination of both upper and lower limbs.

**Figure 1:**
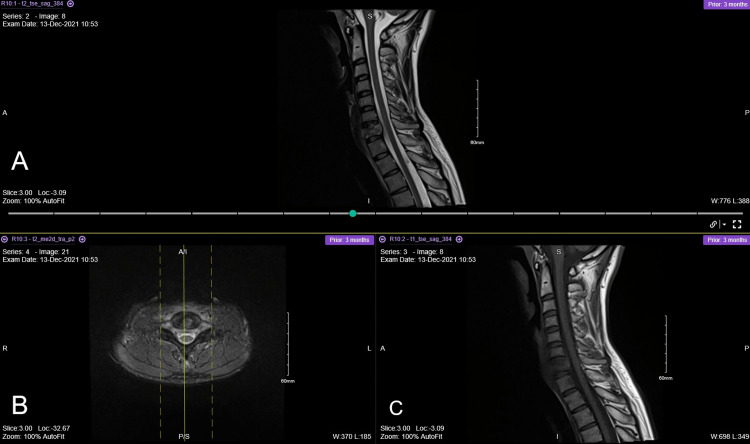
Cervical spine MRI done outside shows C7 destructive lesion with collection size extending from the level of C5 to upper border of T3 anteriorly. (A) T2 sagittal view, (B) axial view, and (C) T1 sagittal view.

**Figure 2:**
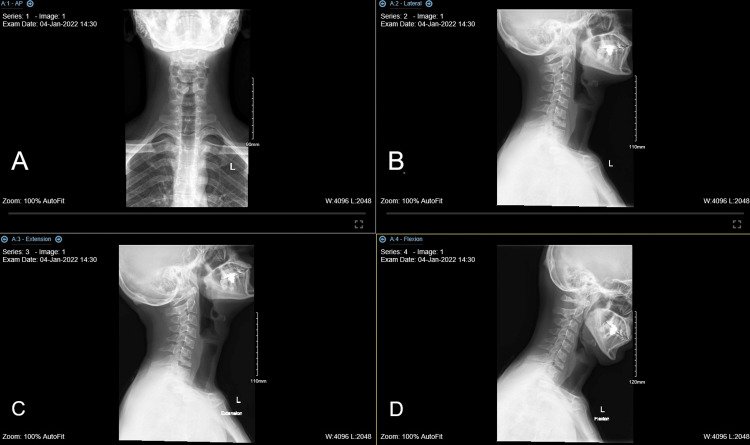
Straightening of the cervical spine is noted. Significant reduction of the vertebral body height of C7 with irregularities of the superior endplate. Narrowing of the disc space of C6-C7 is seen. (A) AP view, (B) lateral view, (C) extension lateral view, and (D) flexion lateral view.

The patient sought medical advice elsewhere prior to his appointment with us and was put on a hard collar. He came to us for a second opinion and management brought the investigations he did, including the magnetic resonance imaging (MRI), outside our institution (Figure 1). Initially, we ordered full blood work, a chest X-ray, consulted the infectious diseases department (ID), did a new orthogonal cervical spine X-ray (Figure 2), as well as computed tomography (CT) planning for biopsy. He had mildly elevated inflammatory markers. The erythrocyte sedimentation rate (ESR) was 78 mm/hour, and C-reactive protein (CRP) was 17.9 mg/L.

**Figure 3:**
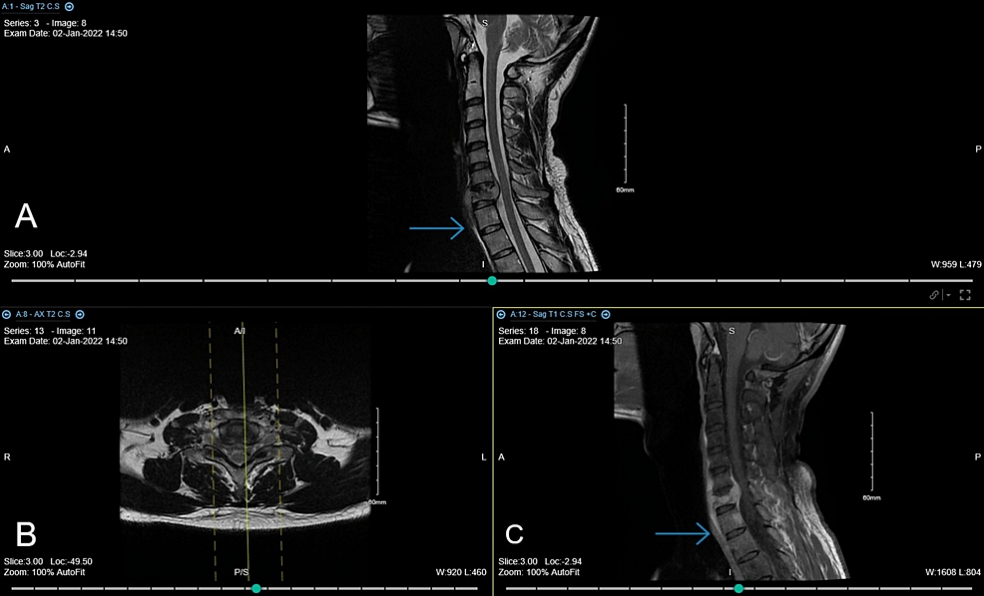
Cervical MRI brought by the patient to the first follow up showed progression of the destruction. Note regression of the retropharyngeal collection size to inferior border of T1. (A) T2 sagittal view, (B) axial view, and (C) T1 sagittal view.

Differential diagnoses included C7 destructive lesions because of TB, malignancy, or due to pyogenic infection. The following visit to the outpatient department one week later revealed that the patient’s complaint progressed to continuous neck and upper back pain that is worse while standing and walking and relieved with lying flat with difficulty maintaining head upright position with moderate left-sided and mild right-sided upper limb numbness and pain for few days prior his follow up. There were no changes in his risk factors, nor were there any new events. The physical exam was still the same. A new MRI, which was done in another center a few days prior to his new follow-up Figure 3, was brought by the patient, which showed progression of the pathological process involving the C7 vertebra, but it oddly showed some regression in the retropharyngeal collection that was seen in the previous MRI, although the patient did not go through any treatment.

**Figure 4:**
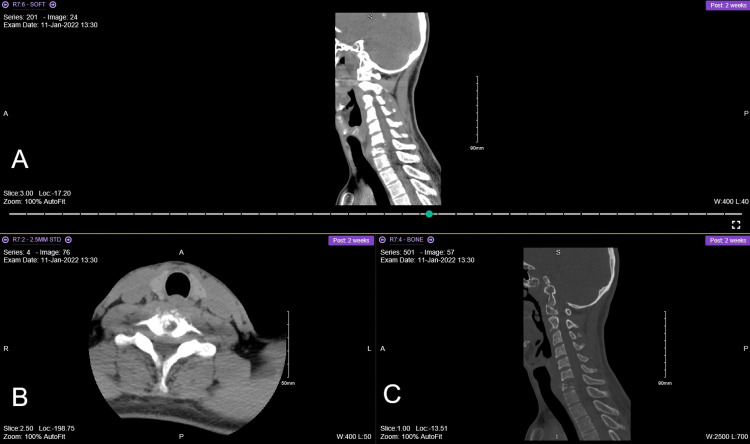
There is straightening with reversed lordosis of the cervical spine. Grade 1 retrolisthesis of C6 over C7. There is reduction in the height of C7 with associated cortical osseous destruction with minimal retropulsion of the upper posterior vertebral body corner. There is minimal facet subluxation. Associated surrounding soft tissue component extending to the prevertebral space and anterior epidural space causing moderate spinal canal stenosis and severe bilateral neuroforaminal stenosis at level C6-C7 and C7-T1. No osseous lesions noted In C6 or T1 vertebral bodies. There is subtle lucency noted in the pedicles of C7. The intervertebral disc spaces are relatively maintained. The craniovertebral junction and clivus are intact. (A) Soft tissue window sagittal view, (B) axial view, (C) bone window sagittal view.

During the above-mentioned visit, the investigations into the patient that were ordered on the previous visit were reviewed. The sputum acid-fast bacillus (AFB) smear was negative. The Mantoux skin revealed only 5 mm of induration. The TB QuantiFERON test was positive. This was conveyed to our colleagues in ID, and it was explained that being an endemic disease in our region, a positive QuantiFERON test might indicate a previous exposure rather than an active infection. Other lab tests were unremarkable, including complete blood count, serology for HIV, thyroid function test, liver function test, and prostate-specific antigen. CT planning for biopsy was done (Figure 4). The interventional radiology department’s opinion after discussion was that the lesion was not approachable by CT-guided biopsy. The patient was added urgently to the next operation list to be operated on within a week.

Four days later, the patient was admitted through the Accidents and Emergency department (A/E). He was complaining of further progression of neck pain and numbness in both hands with no constitutional symptoms. There were also no new events associated with the patient’s complaint to explain his symptoms’ abrupt and unexpected progression. He was afebrile with normal vital signs. A local examination of his neck showed significant tenderness affecting his C-spine with restriction of neck movement. The neurological examination showed reduced power affecting both triceps, which was 4/5, and reduced sensation bilaterally in the index and middle fingers on the right more than the left upper limb with depressed deep tendon reflexes. Other myotomes of the upper limbs were normal and exhibited normal sensation and deep tendon reflexes. The bilateral lower limb exam was normal, and no other positive findings were found on the physical examination.

**Figure 5:**
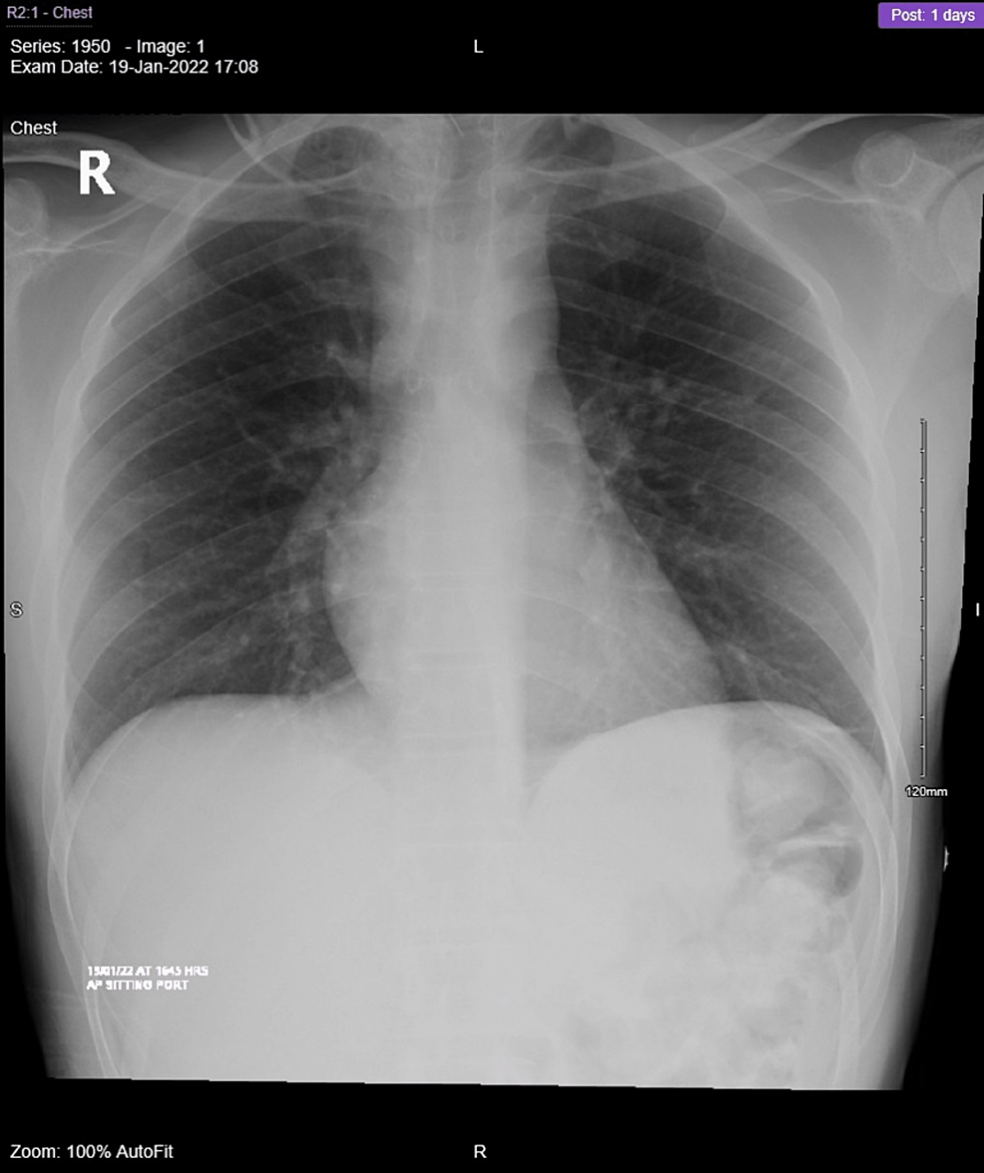
Chest X-ray of the patient. Done during the assessment pre-operative period. It shows no consolidation, pleural effusion, or pneumothorax.

**Figure 6:**
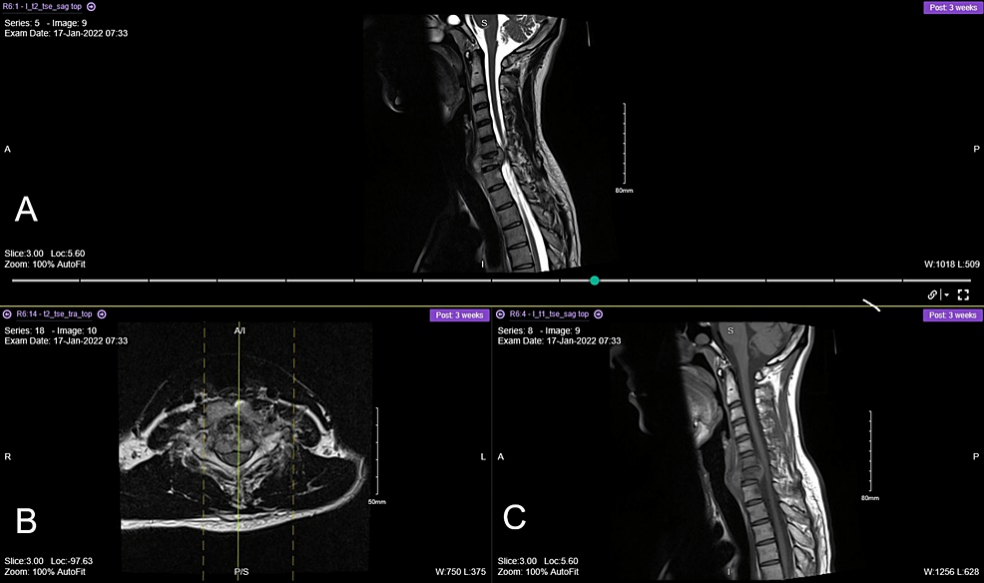
The last (3rd) MRI done for the patient the day before surgery. Showing the progression of the disease. (A) T2 sagittal view, (B) axial view, and (C) T1 sagittal view.

Laboratory evaluation obtained at A/E revealed CRP at 15.1 mg/L and ESR of 63 mm/hour, which displayed a plateauing pattern when compared to the investigations done in the first outpatient department visit. Other laboratory tests were within the reference range. A chest X-ray was done and did not reveal any abnormal findings (Figure 5). Due to the ongoing, rapid, and inexplicable worsening of symptoms, a whole spine MRI with contrast was performed (Figure 6).

The muscle weakness, the severe progression of neck pain, and the new radiological findings led us to operate urgently on the patient on January 18, 2022. This was 14 days after the patient presented to our service on January 4, 2022, with mild axial pain. The patient underwent a C7 biopsy with multiple samples for cultures and an anterior cervical corpectomy and fusion (ACCF) with anterior iliac crest bone grafting.

Intraoperatively, there was a dark yellow, bloody soft tissue mass extending to the spinal canal posteriorly replacing the vertebral body at C7 with no fluid collection, pus, or caseation. Samples taken intra-operatively were investigated. Histopathology was negative for malignancy or neoplastic causes. Intraoperative soft tissue cultures were negative for pyogenic infection. The AFB smear was negative. The Mycobacterium tuberculosis PCR result was weakly positive. ID followed the patient after the results of the PCR and the diagnosis was confirmed as TB spondylodiscitis. The patient was started on anti-tuberculosis drug (ATD) for treatment.

The postoperative course was smooth with no complications. He was started on Cefazolin 1 gram IV every eight hours and a multimodal postoperative pain management protocol, and the drain was removed on the second day postoperatively. There was a significant improvement in his pre-operative symptoms and signs. The patient was discharged five days postoperatively on a hard aspen collar, pain medications, and standard ATD following the recommendations initiated by ID with routine follow-up for dressing and a wound checkup in the orthopedic spine outpatient clinics, which was done one week post-discharge.

**Figure 7:**
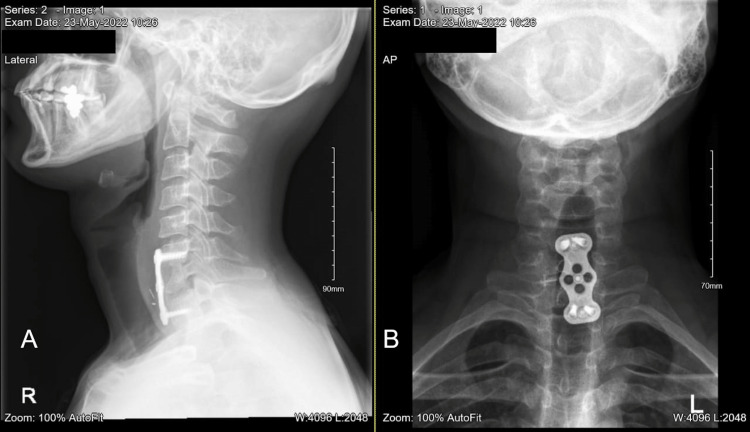
Cervical X-ray four months post-op also shows maintained alignment with signs of union and no signs of hardware complications. (A) Lateral view, (B) AP view.

The patient came in for a routine follow-up six weeks and then four months postoperatively. An AFB culture confirmed positive for Mycobacterium tuberculosis after four weeks of incubation and rifampicin resistance was not detected. A set of cervical X-rays were done, which showed proper hardware position and signs of union (Figure 7). The patient is currently doing well with all symptoms resolved, and there were no signs of neurological sequelae. The surgery treated the patient's pain and progressive symptoms. The patient’s management is still ongoing with ID and orthopedic spine.

## Discussion

The unorthodox progression of this case being a spine TB sheds the light on that not all cases follow the known natural history of the disease. In this particular case, the symptoms of the patient progressed from mild axial and upper back pain to severe pain, difficulty in head upright support, and upper limb weakness over a period of two weeks. The progression was documented on MRI. The patient did not show any constitutional symptoms and his inflammatory markers plateaued during the period of the disease progression below the expected average for such a case.

TB is typically considered a disease of the respiratory system. However, it also presents as extrapulmonary tuberculosis (EPTB), and it accounts for 15-20% of TB-infected patients [9]. Skeletal TB (STB) accounts for 10 % of EPTB, and spinal TB makes up for half of all skeletal EPTB, making it the most common site. The most affected regions, in order, are the thoracic, lumbar, and cervical spine [10,11]. Cervical tuberculosis accounts for 3-5% of all spinal diseases [12].

The typical clinical symptoms of cervical TB include axial or radicular pain, constitutional symptoms, neurological deficit, and deformity. A patient may exhibit axial pain, which accounts for 87% of the pain, or radicular pain, which accounts for 94% of the total pain. Additionally, patients may complain of fever and pressure-related morbidities caused by a retropharyngeal abscess [12]. In the later stages of cervical tuberculosis, it is characterized by severe neck movement restrictions and torticollis [13]. The neurological compromise of cervical TB has been 74% higher than the rest of the spine’s TB. Furthermore, the neurological deficit associated with cervical TB can be triggered by mechanical compression resulting from pathological fractures and spinal instability, primary cord involvement, and thrombosis. Research has indicated that neurological deficit incidences are more prevalent in adults, as high as 81%, mainly influenced by factors such as spinal rigidity [12]. Lesions due to TB generally affect the anterior vertebral bodies, leaving the posterior elements unaffected [14].

Clinical diagnosis for cervical TB, aided by heightened suspicions, comprises laboratory workup, imaging, and tissue diagnosis [5]. Laboratory work entails blood investigations and immunological tests. In blood investigations, ESR and CRP are the main parameters. In two-thirds of individuals with spinal tuberculosis, higher ESR and CRP have been witnessed. It must be noted that persistent elevated ECR and CRP beyond three months of ATD treatment triggers suspicion of drug resistance or insufficient dosage [12]. Our patient’s inflammatory markers, ESR and CRP, demonstrated a state of ongoing inflammation. However, the rapid and severe deterioration of the patient's condition, which was displayed clinically and radiologically, did not display the severity with inflammatory markers, even though the patient was immunocompetent. It has been reported that ESR can be as high as 145 mm/h [15], and CRP can also be as high as 197 mg/l for patients diagnosed with spinal TB [16].

The imaging modality of choice is MRI to detect the disease in its earliest stages. Also, the growth of mycobacterium TB from infected tissue is considered the gold standard for TB diagnosis [5]. The tissue sampling approach entails the analysis of sample tissues acquired from the infected foci. It comprises techniques such as polymerase chain reaction, bacterial culture, tubercular culture, and histopathological examination [12].

As for the immunological tests, interferon-gamma (IFN-γ) release assays are enzyme-linked immunosorbent assay tests that measure the amount of IFN-γ released by the immune system against TB antigens. The use of immunological tests can be limited in endemic regions such as Saudi Arabia. Cervical TB's typical presentation includes para-discal and central presentation [12].

**Figure 8:**
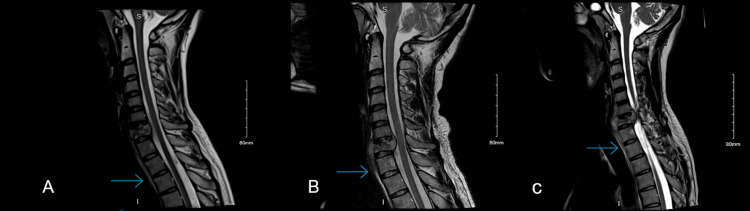
(A) Initial MRI imaging showing C7 destruction and collection extending to upper border of T3. (B) This is the second MRI brought by the patient at follow-up less than 3 weeks after the first one, in which despite ongoing destruction we can see regression in the size of the collection to the inferior border of T1. (C) MRI done pre-op which was done about 2 weeks after, which showed further destruction and stenosis of the spinal canal, however the collection size remained almost similar to the follow up MRI.

The patient’s radiological findings distinctly visualized the unusual rapid deterioration, within around two-week intervals, through three consecutive MRIs. We were able to see, at first, the collection formation extending from the level of C5 to T3, noting the peculiar regression in size with no treatment, in addition to the spinal canal stenosis growing narrower as we strolled through the MRIs, which eventually caused a mass effect on the spinal cord (Figure 8).

ATD remains the basis of the management of spinal TB. Isoniazid, rifampicin, ethambutol, pyrazinamide, and streptomycin are the first-line ATDs used for the treatment. The primary factor that determines the success of conservative treatment with ATD is the organism’s sensitivity towards it. In accordance with recent guidelines from WHO, a conventional regimen consists of two phases: four drugs for the duration of two months as the intensive phase, and two or three drugs for seven to nine months as the continuation phase. Surgical intervention is indicated, especially when tissue sampling is required, and also whenever spinal decompression, debridement, or stabilization is needed [12].

## Conclusions

Since tuberculosis is common in Saudi Arabia and can present in several forms, cervical TB should be considered in the differential diagnosis of any patient presenting with a cervical spine lesion without relying much on the expected slow course of skeletal TB. Our patient's symptoms progressed severely within a period of two weeks, which was also documented on MRI. In addition, no constitutional symptoms were evident by the patient; his inflammatory markers plateaued during the period of the disease progression below the expected average for such a case. This shows the significance of shedding light on this unanticipated and quick deterioration of the clinical manifestation of a known disease.
